# Spontaneous Coronary Dissection: “Live Flash” Optical Coherence Tomography Guided Angioplasty

**DOI:** 10.1155/2016/5643819

**Published:** 2016-02-17

**Authors:** Angela Pimenta Bento, Renato Gil dos Santos Pinto Fernandes, David Cintra Henriques Silva Neves, Lino Manuel Ribeiro Patrício, José Eduardo Chambel de Aguiar

**Affiliations:** Hospital do Espirito Santo, Largo Senhor da Pobreza, 7000-811 Évora, Portugal

## Abstract

Optical Coherence tomography (OCT) is a light-based imaging modality which shows tremendous potential in the setting of coronary imaging. Spontaneous coronary artery dissection (SCAD) is an infrequent cause of acute coronary syndrome (ACS). The diagnosis of SCAD is made mainly with invasive coronary angiography, although adjunctive imaging modalities such as computed tomography angiography, IVUS, and OCT may increase the diagnostic yield. The authors describe a clinical case of a young woman admitted with the diagnosis of ACS. The ACS was caused by SCAD detected in the coronary angiography and the angioplasty was guided by OCT. OCT use in the setting of SCAD has been already described and the true innovation in this case was this unique use of OCT. The guidance of angioplasty with live and short images was very useful as it allowed clearly identifying the position of the guidewires at any given moment without the use of prohibitive amounts of contrast.

## 1. Introduction

Optical Coherence tomography (OCT) is a light-based imaging modality which shows tremendous potential in the setting of coronary imaging. Compared to intravascular ultrasound (IVUS), OCT has a tenfold higher image resolution. It has the ability to characterize the structure and extent of coronary disease with unprecedented detail as the various components of atherosclerotic plaques have different properties. In daily practice OCT is useful in guiding complex interventions. Image acquisition has some particularities because blood must be displaced during OCT imaging. In fact OCT images are obtained and recorded as the coronary artery is flushed with contrast and the catheter-imaging tip is pulled back (usually at 20 mm/s). Spontaneous coronary artery dissection (SCAD) is an infrequent cause of acute coronary syndrome (ACS) typically affecting a younger, otherwise healthy population. The population-based incidence of SCAD is unknown. Retrospective registry studies have reported SCAD detection in 0.07% to 1.1% of all coronary angiograms performed. Apparently there is a female preponderance and an association with peripartum or postpartum status. Other identified SCAD associations include connective tissue disorders, vasculitis, polycystic kidney disease, and exercise, suggesting an underlying vascular predisposition in some, although a unifying structural vessel wall abnormality has not yet been identified [1, 2]. The diagnosis of SCAD is made mainly with invasive coronary angiography, although adjunctive imaging modalities such as computed tomography angiography, IVUS, and OCT may increase the diagnostic yield [3]. OCT provides unique insights on the most relevant morphologic features of the condition including entry tear, intimomedial flap, double-lumen morphology, intramural hematoma, and associated thrombus [4]. The optimal treatment strategy for acute SCAD presentation remains undetermined and may vary according to the type and severity of presentation. Reports have demonstrated favorable outcomes with conservative management (with documented angiographic resolution), fibrinolysis, percutaneous coronary intervention (PCI), and coronary artery bypass grafting (CABG). Regardless of initial treatment strategy, in-hospital and early outcomes have in general been reported to be favorable.

## 2. Case Report

A 41-year-old female with obesity and polycystic kidney disease is referred to the cath lab after an episode of chest pain with troponin elevation one week after primary PCI in the context of inferior acute myocardial infarction with ST elevation. Angiography at the time revealed a balanced-dominant circulation, with TIMI flow 3 in the right coronary artery (RCA) without stenotic lesions and a subocclusive lesion in a small posterolateral branch (PLB) from the circumflex artery which was considered to be the culprit lesion. Balloon angioplasty was performed with good final result and TIMI 3 flow.

**Figure 1:**
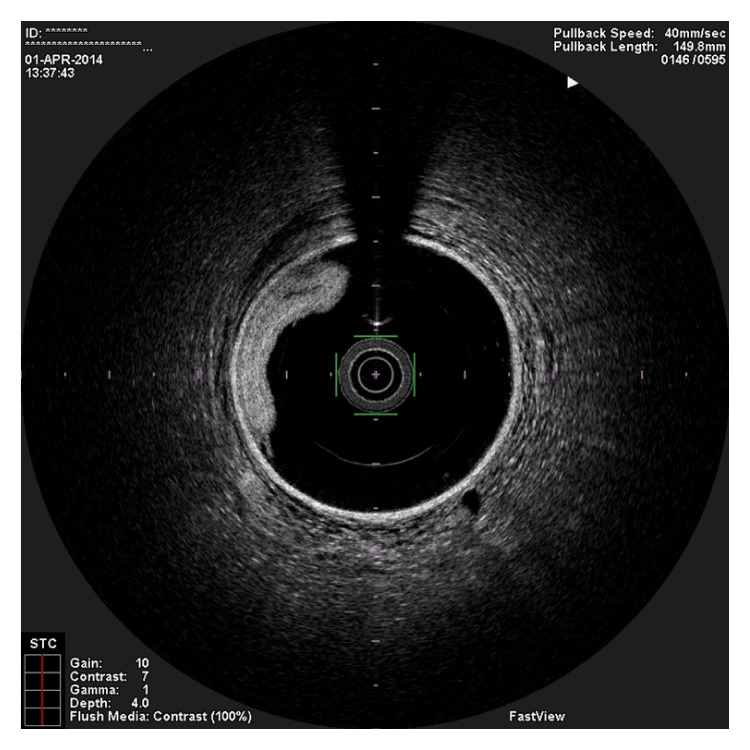
OCT image showing the guidewire in the false lumen as the true lumen is crushed against the vessel wall.

**Figure 2:**
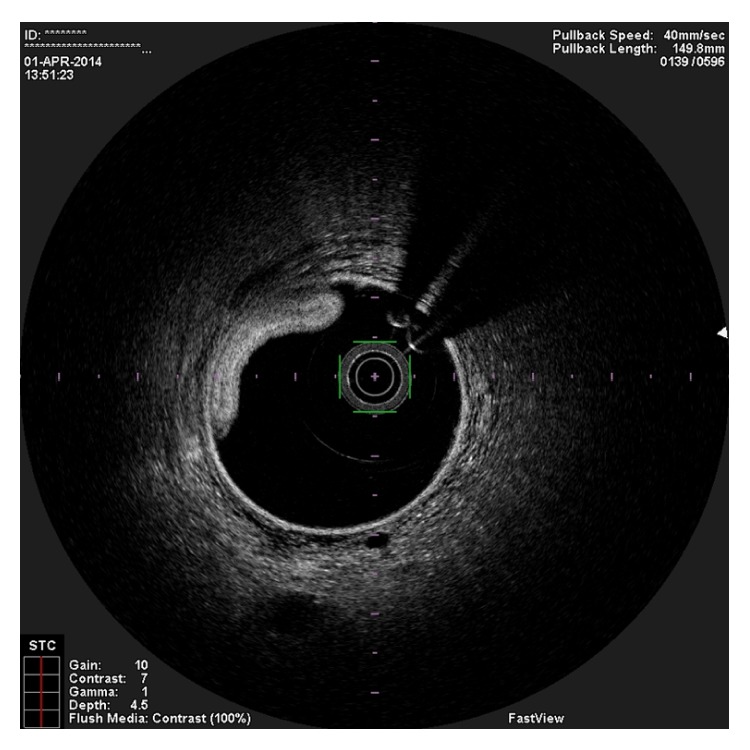
OCT image showing two guidewires in the false lumen.

**Figure 3:**
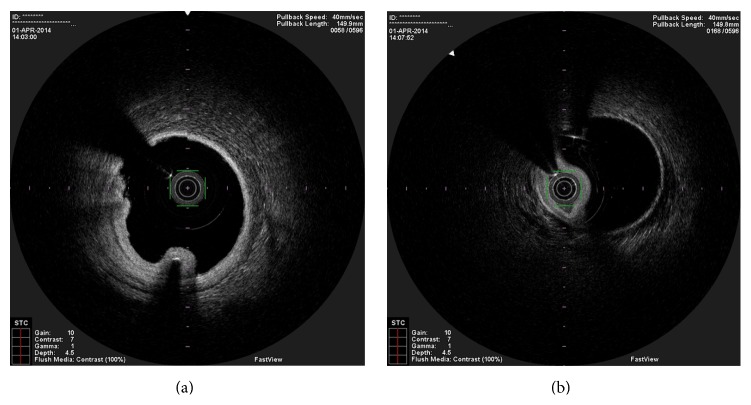
OCT image showing one guidewire in the false lumen and the other in the true lumen.

**Figure 4:**
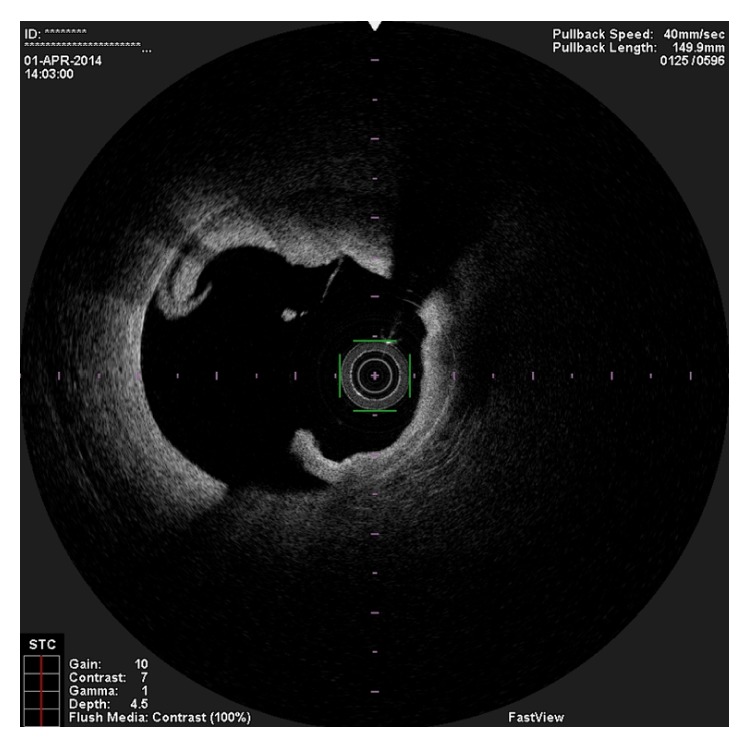
OCT image showing the entry tear of the spontaneous dissection.

**Figure 5:**
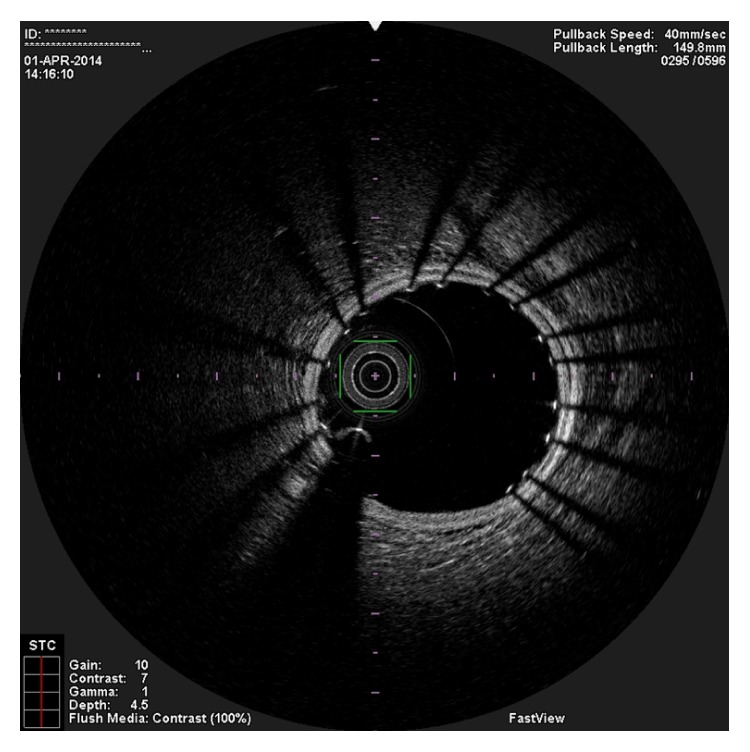
OCT performed after stent implantation showing good apposition.

**Figure 6:**
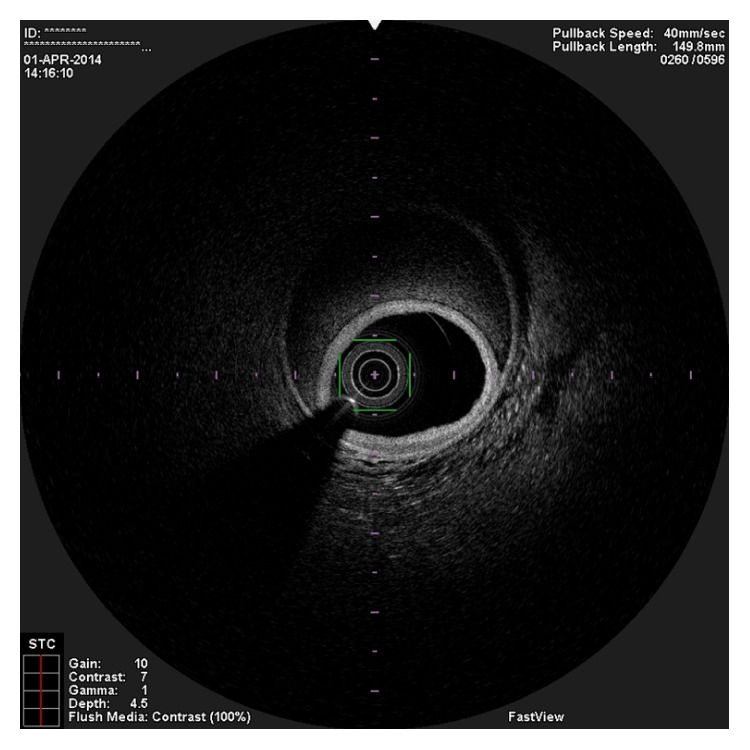
OCT image showing the guidewire in the true lumen and the false lumen with signs of thrombosis in process.

**Figure 7:**
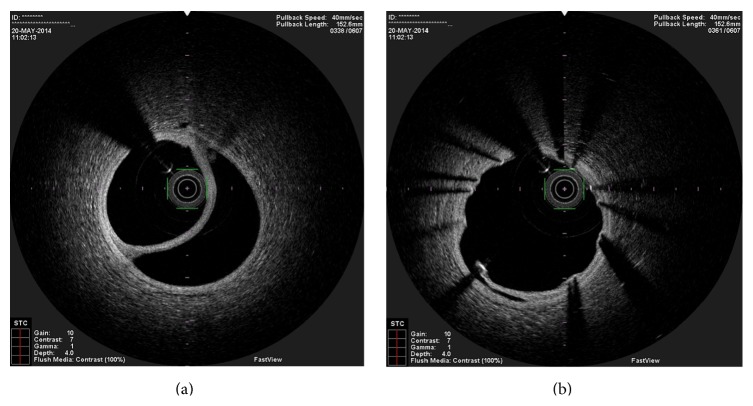
OCT performed one month after the stent angioplasty, showing the maintenance of the dissection. (a) shows clearly that the guide wire is in the true lumen.

**Figure 8:**
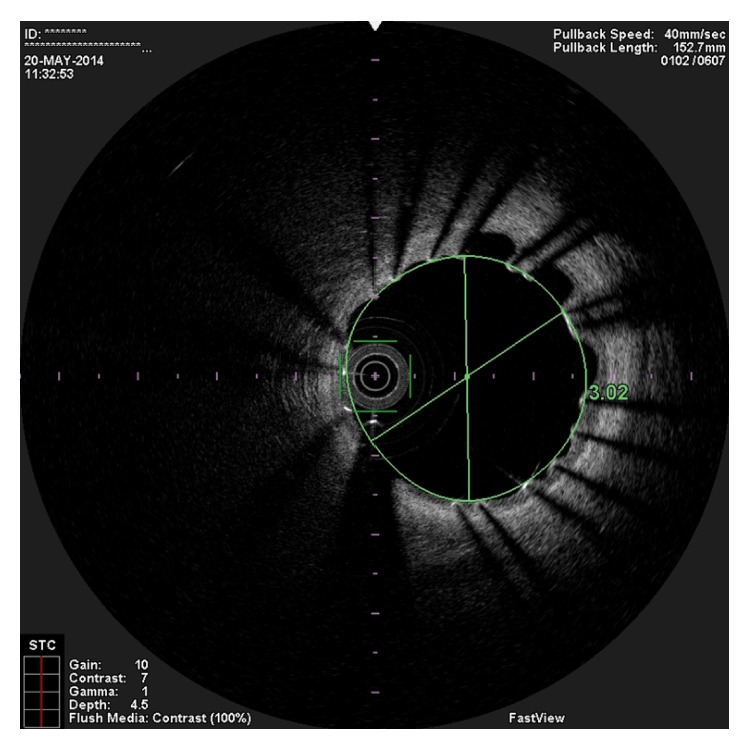
OCT performed after implantation of additional stents in medial and distal RCA and the balloon after dilatation.

In the present episode angiography showed persistent good result of the previous PCI, and total occlusion of the proximal RCA, that was suspected to be caused by spontaneous dissection because of the presence of haziness at the site of the occlusion and disappearance of several acute marginal branches as compared with the previous exam. We then decided to perform OCT (Terumo Lunawave® OFDI system) that clearly revealed the double-lumen morphology and also that the guidewire was in the false lumen (Figures 1 and 2). We then proceeded to angioplasty under OCT guidance. With live OCT images, without recording an actual pullback, we used small flushes of contrast until we were able to confirm that the guidewire was on the true lumen (Figures 3(a) and 3(b)). The OCT could also identify entry tear very clearly (Figure 4). Once the true lumen was secured, we proceeded to angioplasty with a drug-eluting stent sealing the entry tear in the proximal right coronary artery (Figure 5). There was final TIMI 3 flow, and the acute marginal branches were again visible, but there was a narrowing in the distal vessel where the false lumen was still visible but showing signs of thrombosis (Figure 6). To avoid making a full metal jacket, we decided to accept this result and perform a control angiography one month later. After one uneventful month, follow-up angiography revealed persistence of a large dissection that extended to the distal vessel. There was an important compromise of the true lumen by the false lumen. Given the previous STEMI presentation and the negative angiographic evolution within a month, we carried out an OCT that confirmed the dissection and showed that the guidewire was now in the true lumen (Figures 7(a) and 7(b)). In this setting, we decided to seal the dissection with implantation of two more drug-eluting stents in the mid and distal RCA to guarantee the long-term patency of the vessel (Figure 8). There was residual dissection in the very distal segment, which was a small vessel.

## 3. Discussion

Diagnosis and management of SCAD is very challenging. However, an accurate and early diagnosis remains of paramount importance. There are few data concerning the utilization and value of OCT in this rare disease. In the case cited before, OCT was able to readily visualize the double-lumen morphology characteristic of this entity and to identify the entry tear and the circumferential nature and longitudinal extent of the disease. The compromise of the true lumen and the distribution of the false lumen were also clearly visualized. The low profile (catheter crossing profile: 2,6 Fr) and higher resolution (frame rate: 160 fps) of OCT were very useful in this case as they allowed obtaining high quality images of long segments of the artery. OCT is not, however, without potential harm. In a situation of a SCAD, it can further propagate the dissection and/or intimal tear, due to brisk intracoronary flow changes, so its use must take into account the potential benefit versus potential harm. Careful contrast injection speed is of paramount importance. In this case OCT was revealed to be particularly useful, since not only did it make it possible to correctly identify the dissection and intimal tear, it also made correct true lumen wiring possible, with live imaging during wire manipulation with a stationary OCT catheter. OCT usefulness in the setting of SCAD has already been reported, and the technique we describe in this case represents an innovation that may become even more interesting in the future, with OCT technical development. This technique opens a promising field for software development; for example, we think that it will be very useful to be able to also record images with a stationary OCT catheter and not only those obtained in the automated pullback with complete flush of the coronary artery. Another potential benefit of this technique (OCT) in SCAD setting is the possibility of doing 3D OCT online to improve the capability of identifying the entry tear. Regarding the angioplasty per se, we would have used bioresorbable scaffolds, were they available in our cath lab at the time of this procedure. In a more detailed analysis of the OCT images after the final procedure we realized that the entry tear was located behind the distal part of the previously implanted stent (Figure 7(b)). We now wonder if we could have sealed the dissection with just a balloon after dilatation.

## 4. Conclusion 

Spontaneous coronary artery disease is an intriguing entity, in which a conservative approach seems to be the best option whenever possible, due to procedural complications. OCT can be decisive for correct diagnosis, planning, treatment, and optimization of PCI in SCAD.
